# Supplementary material for: Meta-Analysis and Gene Set Enrichment Relative to ER Status Reveal Elevated Activity of MYC and E2F in the “Basal” Breast Cancer Subgroup
Source: PLoS One. 2009 Mar 9;4(3):e4710. doi: 10.1371/journal.pone.0004710 (PMC2650420; doi:10.1371/journal.pone.0004710)
Supplement: Dataset S1 — GSEA results from first screen. The file contains a zipped archive of the HTML results of the second GSEA screen. Reports for enriched or depleted datasets may be accessed from the “index.html” link within the my_analysis.GseaPreranked.1219975950625.rpt.zip ZIP archive. Enrichment plots are available for each of the 50 most enriched and depleted gene sets. (13.32 MB ZIP) [file pone.0004710.s001.zip › my_analysis.GseaPreranked.1219975950625.rpt.zip!//CHR17Q24.html]

Details for gene set CHR17Q24[GSEA]

|  || Dataset | zSco.ERneg.to.ERpos\_collapsed |
| Phenotype | NoPhenotypeAvailable |
| Upregulated in class | na\_neg |
| GeneSet | CHR17Q24 |
| Enrichment Score (ES) | -0.58518136 |
| Normalized Enrichment Score (NES) | -2.0150151 |
| Nominal p-value | 0.002915452 |
| FDR q-value | 0.008298658 |
| FWER p-Value | 0.167 |
Table: GSEA Results Summary

  

Fig 1: Enrichment plot: CHR17Q24      
 Profile of the Running ES Score & Positions of GeneSet Members on the Rank Ordered List

  

| PROBE | GENE SYMBOL | GENE\_TITLE | RANK IN GENE LIST | RANK METRIC SCORE | RUNNING ES | CORE ENRICHMENT || 1 | PPM1E |  |  | 4788 | 1.226 | -0.3311 | No |
| 2 | RGS9 |  |  | 4807 | 1.220 | -0.3036 | No |
| 3 | CDC42EP4 |  |  | 5405 | 1.009 | -0.3246 | No |
| 4 | PSMD12 |  |  | 5876 | 0.852 | -0.3397 | No |
| 5 | CCDC46 |  |  | 5902 | 0.847 | -0.3216 | No |
| 6 | GNA13 |  |  | 6893 | 0.521 | -0.3837 | No |
| 7 | PITPNC1 |  |  | 7283 | 0.390 | -0.4037 | No |
| 8 | ACOX1 |  |  | 7683 | 0.237 | -0.4281 | No |
| 9 | CACNG5 |  |  | 8269 | 0.010 | -0.4719 | No |
| 10 | MBTD1 |  |  | 8401 | -0.039 | -0.4808 | No |
| 11 | GRIN2C |  |  | 8536 | -0.097 | -0.4886 | No |
| 12 | PRPSAP1 |  |  | 8951 | -0.285 | -0.5130 | No |
| 13 | NOL11 |  |  | 9190 | -0.400 | -0.5214 | No |
| 14 | GRB2 |  |  | 9586 | -0.610 | -0.5367 | No |
| 15 | HELZ |  |  | 10201 | -0.958 | -0.5601 | Yes |
| 16 | CACNG1 |  |  | 10225 | -0.973 | -0.5388 | Yes |
| 17 | SSTR2 |  |  | 10708 | -1.321 | -0.5438 | Yes |
| 18 | FALZ |  |  | 11259 | -1.793 | -0.5427 | Yes |
| 19 | LLGL2 |  |  | 11821 | -2.348 | -0.5293 | Yes |
| 20 | CACNG4 |  |  | 11858 | -2.378 | -0.4757 | Yes |
| 21 | WIPI1 |  |  | 12116 | -2.722 | -0.4306 | Yes |
| 22 | AMZ2 |  |  | 12335 | -3.003 | -0.3759 | Yes |
| 23 | ABCA8 |  |  | 12430 | -3.149 | -0.3084 | Yes |
| 24 | SLC9A3R1 |  |  | 13043 | -4.624 | -0.2449 | Yes |
| 25 | FDXR |  |  | 13054 | -4.663 | -0.1352 | Yes |
| 26 | SLC16A6 |  |  | 13298 | -6.552 | 0.0017 | Yes |
Table: GSEA details [plain text format]

  

Fig 2: CHR17Q24: Random ES distribution      
 Gene set null distribution of ES for **CHR17Q24**
